# Teaching punch biopsy and suturing with a 3D-printed skin model: design and integration into the medical curriculum

**DOI:** 10.1186/s41205-026-00317-x

**Published:** 2026-02-24

**Authors:** Sandra Schuh, Stefan Schiele, Anna Rubeck, Ludwig Christian Hinske, Julia Welzel, Alexander Schneller

**Affiliations:** 1 Department of Dermatology and Allergology, University Hospital Augsburg, Sauerbruchstr. 6, 86179 Augsburg, Germany; 2 Faculty of Medicine, Medical Didactics and Educational Research, University of Augsburg, DEMEDA, Augsburg, Germany; 3 Institute of Digital Medicine, University Hospital of Augsburg, Augsburg, Germany; 4 Chair of Computational Statistic and Data Analysis, Institute of Mathematics, University of Augsburg, Augsburg, Germany

**Keywords:** 3D skin model, Dermatology, Training, Education, Teaching, Punch biopsy, Suturing, 3D technology, 3D printing, 3D modeling

## Abstract

**Background:**

In the 2019/2020 winter semester, the University of Augsburg’s Faculty of Medicine introduced a competence-oriented model degree program with a spiral curriculum integrating theory and practice. A key feature, the clinical longitudinal course, emphasizes practical skills such as skin examination. Existing training materials for punch biopsies, e.g., foam models and fruit, have proven insufficient. This project aimed to create a realistic, cost-effective, reusable three-dimensional (3D) skin model to improve the teaching of punch biopsy and suturing techniques.

**Methods:**

The 3D skin model was developed in a multistage process. It began with a 3D scan created via a handheld 3D scanner and refined in 3D modeling software. A fused deposition modeling (FDM) printer produced negative molds that were filled with silicone, resulting in a realistic model. After several iterations, a design was achieved that successfully simulated the tactile and functional aspects of punch biopsy and skin suturing. Student feedback was collected through an anonymous online questionnaire assessing perceived realism, usefulness for practicing punch biopsies and suturing, and impact on their confidence.

**Results:**

The silicone-based skin simulator debuted in the 2023–2024 winter semester’s ‘examination of the skin’ course. A total of 82 students participated in the course, of whom 58 completed the evaluation questionnaire. The students used the model to perform punch biopsies and suturing, reporting that its material properties allowed these procedures to be practiced under course conditions. With a low production cost (of 0.62 € per model) compared to commercial models, it is a cost-efficient alternative to previous materials. The students provided positive feedback, reporting increased confidence in performing these procedures on humans for the first time.

**Conclusions:**

The 3D training model is an important advancement in introducing 3D technologies in practical training, providing realistic, cost-effective practice for punch biopsy and suturing. Its successful integration into the curriculum highlights its potential for broader applications in medical education. The evaluation indicated that the model provided realistic skin properties and proved effective for practicing punch biopsies and suturing, thus addressing the limitations of traditional training materials.

## Introduction

The Medical Faculty of the University of Augsburg trains medical students through a competency-based model curriculum introduced in 2019. Central to this approach is the early linkage of theoretical knowledge with practical application. The ultimate goal is to prepare students for independent medical practice and ongoing professional development, as outlined in the National Competency-Based Learning Objective Catalog (NKLM 2.0, 2021) [1, 2].

Augsburg’s program emphasizes the acquisition of clinical skills through modular and longitudinal courses, which are supported by theoretical sessions, and culminates in clinical internships where students apply and refine their competencies in real-world settings. Within this context, the Department of Dermatology identified performing a diagnostic skin biopsy with single interrupted sutures as a critical skill. Such procedures are frequently required during clinical clerkships and internships. Within this curriculum, technically supported simulation plays a central role in enabling students to link theoretical knowledge with procedural competence at an early stage. The newly developed 3D skin model was therefore conceived not only as a technical prototype, but as a structured learning tool embedded in the dermatology skills course. It directly addresses competency-based learning objectives related to performing diagnostic punch biopsies and basic wound closure, which are key components of the NKLM 2.0 and essential for early clinical training. Simulation-based medical training is essential for acquiring these practical skills before they are applied to actual patients [3]. Research has shown that simulations not only improve learner confidence and competence but also enhance patient safety in recent clinical training environments [4–7]. Suturing, in particular, is a fundamental skill for physicians across specialties and is necessary to minimize complications, such as infection or excessive scarring, while promoting faster wound healing and better cosmetic outcomes [8].

The skin models used for training can be broadly categorized into low-fidelity and high-fidelity models, each with distinct advantages and disadvantages [9]. The term fidelity primarily refers to the realism of the model or simulator in comparison to human patient skin [8, 10]. Low-fidelity models include synthetic materials such as silicone mats or foam and organic materials such as fruit skins (e.g., banana or orange skins) [9–13]. These models are affordable, widely available, portable, and reusable without supervision [9–14]. However, they often lack the tactile realism required to mimic human skin accurately. High-fidelity models, such as animal tissues (e.g., chicken skin, pig feet) or human cadavers, provide a more realistic simulation of human skin [9, 15–17]. Despite their advantages, these models are costly, require ethical clearance, pose infection risks, and demand specialized storage conditions [8, 9].

Studies have demonstrated that low-fidelity models can be nearly as effective as high-fidelity models for teaching basic suturing techniques [18, 19]. For example, fruit skins are frequently used as substitutes, with banana skins showing particular promise for simple sutures such as single interrupted and vertical mattress techniques [19, 20]. However, more complex procedures, such as subcutaneous sutures, require higher-fidelity models such as pig skin or advanced synthetic alternatives [19]. The high cost of commercially available models, combined with the growing number of students requiring training, has prompted the search for more accessible and realistic alternatives [21, 22]. In addition, the validation of these silicone suture trainers by medical professionals and learners is usually lacking [14]. A 2016 survey of medical students in England revealed that 86% (526 out of 705 students from 16 medical schools) found their practical suturing training inadequate [23]. Furthermore, 44% reported not meeting the competency standards for a simple interrupted suture, and 84% reported not meeting the competency standards for a subcutaneous suture [23]. Notably, 20% of the students stated that they had paid for additional suturing courses [23].

For this reason, we consider it essential to ensure practical teaching and continuous improvement in skills related to performing skin biopsies and subsequent suturing. This goal could be achieved by providing more affordable, accessible, anatomically accurate, and equivalent alternatives to animal and synthetic skin materials [3]. Similarly, Bartellas et al. emphasized that tactile three-dimensional (3D) models can be useful in education by helping participants better understand anatomy, enhancing their training skills, and building confidence [3, 24]. However, few to no 3D-printed skin simulators are currently available for the practical training of medical students, with slightly more options available for the advanced training of residents [25–27].

This paper aims to address the limitations of existing skin models by introducing a cost-effective, realistic 3D-printed skin model developed for teaching punch biopsy and suturing techniques. The model mimics human skin with anatomically accurate features and provides a practical alternative to expensive or less realistic training materials. By incorporating this model into Augsburg’s clinical curriculum, the project aims to increase the quality and accessibility of hands-on medical training, ensuring that students are better prepared for clinical practice.

## Materials and methods

The aim of this study was to develop and evaluate a cost-effective 3D-printed skin model for teaching punch biopsy and suturing. The project was designed as a single-center, exploratory, prospective observational pilot study embedded within a curriculum-integrated educational evaluation at the University of Augsburg during the winter semester of 2023–2024. The implementation of the model was situated within the mandatory second-year skills course ‘Examination of the Skin’. The teaching sequence consisted of a preparatory theoretical unit followed by supervised practical stations in which students performed punch biopsies and sutures directly on the model. This structured integration ensured that the simulator complemented the existing curriculum rather than functioning as an isolated technical exercise. To ensure a systematic development process, the procedural steps reproduced in the model were based on established dermatologic biopsy guidelines and standard teaching materials used in our curriculum. These included institutional procedural manuals, NKLM 2.0 competency requirements, and widely accepted clinical descriptions of punch biopsy techniques. The selection of procedural steps followed the sequence typically taught to medical students: lesion assessment, disinfection, sterile draping, local anesthesia, verification of adequate anesthesia, punch placement, excision depth control, hemostasis, subsequent wound closure using single interrupted sutures and final dressing application.

The project was conducted as a curriculum-integrated educational development and evaluation study within a mandatory undergraduate dermatology skills course. It was designed to gather anonymized student feedback to support the development and refinement of a teaching intervention and did not involve any clinical intervention or patient-related outcomes.

Ethical approval for the evaluation of student feedback was obtained from the Ethics Committee of the Ludwig Maximilian University of Munich (project number 23–0884). In addition, separate ethical approval was granted for the creation of the 3D facial scan used for model development (project number 23–0536).

Due to the exploratory educational nature of the project and the absence of patient-related data or health outcomes, formal study registration in a clinical trial registry was not required.

### Creation of a 3D scan

**Fig. 1:**
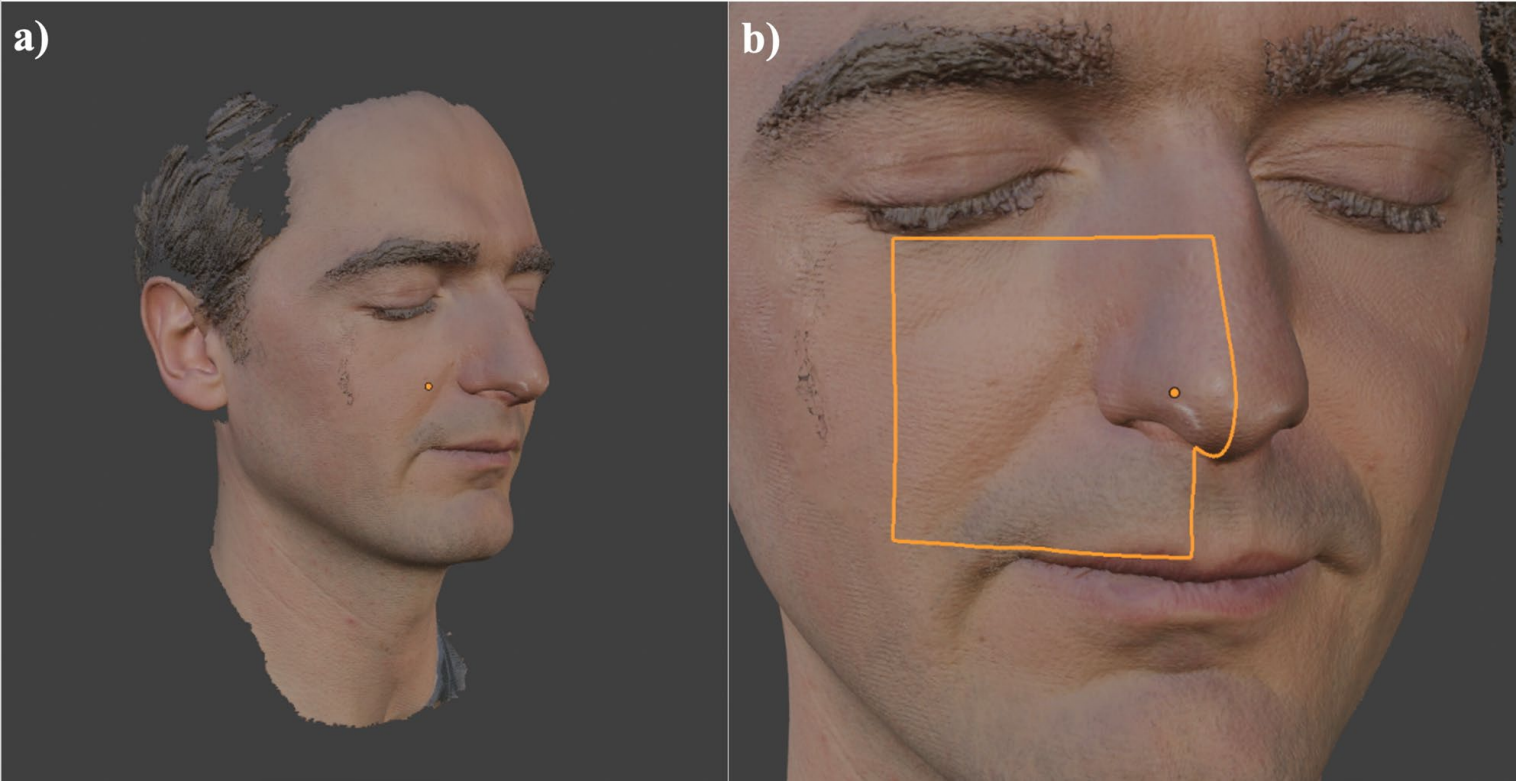
Workflow from a 3D scan to a printable model. (**a**) 3D scan of a face used as a template taken with the Artec Space Spider 3D scanner. (**b**) Selection of the anatomical region (cheek, nasal ala, nasolabial fold)

The starting point for creating a dermatological biopsy simulator is the 3D model of the required structure. This model can be generated in several ways, such as through 3D modeling without a physical template, akin to shaping a ceramic sculpture from clay, or via a 3D scan. In this instance, a 3D scan of a face was used as the template for further modeling (Fig. 1a). For privacy reasons, the scan was performed with the consent of a dermatological resident, as 3D data are considered sensitive biometric information. Additionally, positive ethics approval (project number 23–0536) was obtained from Ludwig Maximilian University of Munich for the creation of the 3D model.

Laser triangulation and structured-light devices are among the most widely used scanning technologies. Structured light devices have the advantage of not emitting radiation harmful to the eyes, making them particularly suitable for use with patients. For this project, the Artec Space Spider (Artec 3D, Senningerberg, Luxembourg), a device utilizing structured-light technology, was employed.

### Conversion of the 3D scan into a printable model

The model created with the 3D scanner (Fig. 1a) is subsequently loaded into a 3D modeling suite. A powerful and free option for this process is Blender (Blender Version 4.1.0, Blender Foundation – www.blender.org, General Public License (GPL)). The first step involves selecting the specific area relevant to the intended simulator. In this case, the particularly intricate region of the cheek, nasal ala, and nasolabial fold was chosen (Fig. 1b) because these subunits are among the most common sites of nonmelanoma skin cancer, especially basal cell carcinoma, and therefore commonly require biopsy [28, 29]; given their central location within facial aesthetic units, procedures in this region warrant special attention with respect to cosmetic outcomes [30, 31]. This anatomical selection was chosen by clinical frequency data and teaching relevance, as these regions represent common biopsy sites in dermatology and thus align closely with procedural steps students must master early in training.

**Fig. 2:**
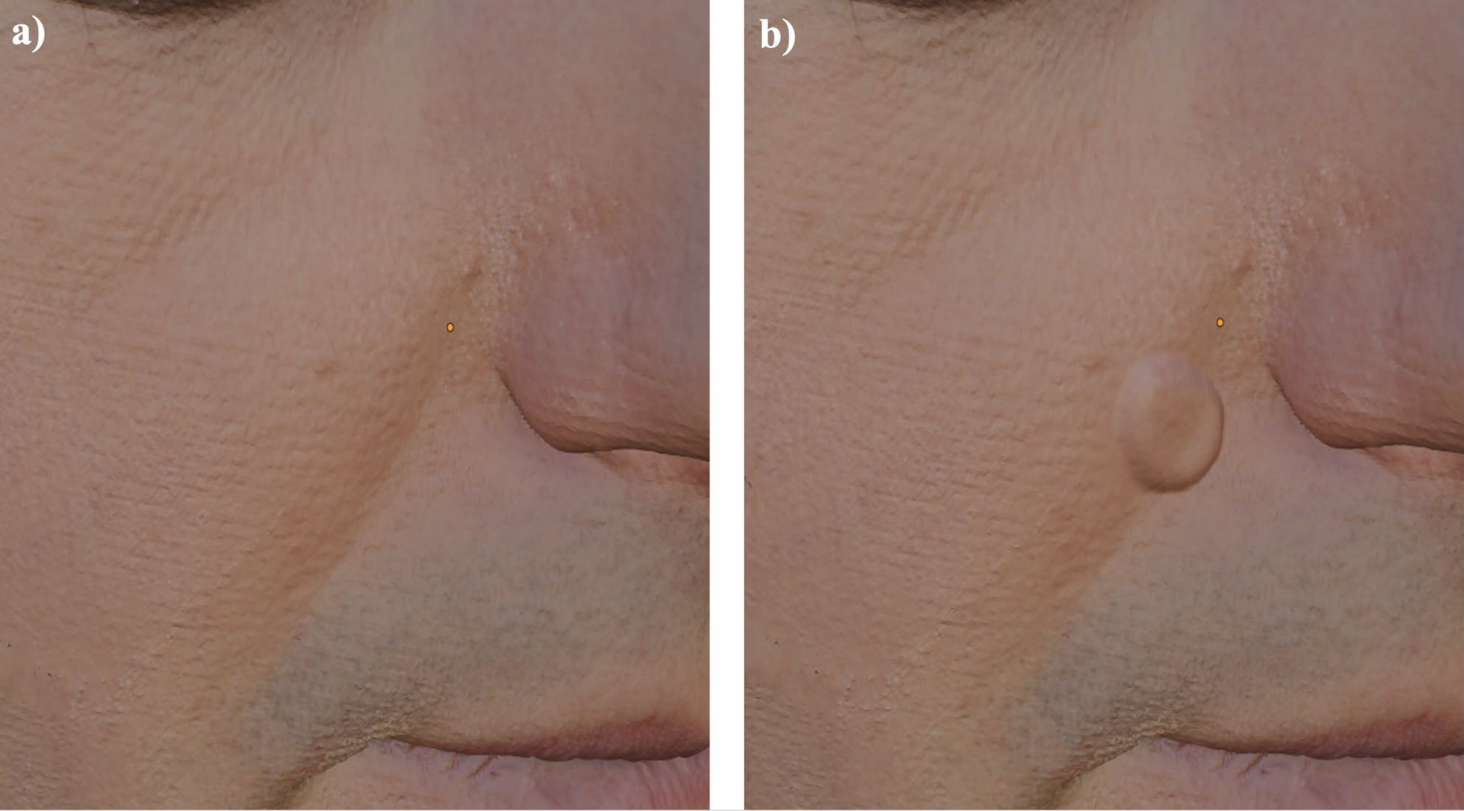
Sculpting of pathological structures. Creation of lesions in Blender using sculpt mode. (**a**) shows the skin model without (**b**) with a suspicious lesion for basal cell carcinoma

**Fig. 3:**
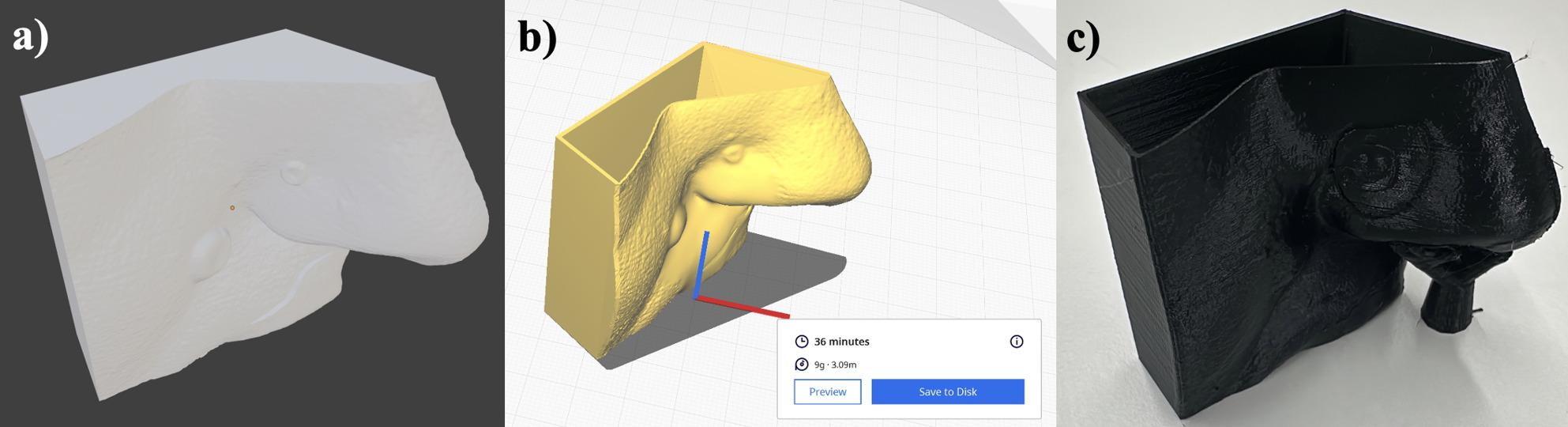
Preparation of 3D printing. (**a**) Color omitted during design due to silicone reproduction. (**b**) Model prepared with Cura slicer software. (**c**) Printed mold before silicone casting

In this selected area, pathologies to be used later in the simulation are created via various 3D tools, particularly those available in the ‘sculpt mode’ of Blender (Fig. 2a and b). Flat air channels were used to simulate the presence of multiple skin layers for mobilization. Since the planned artificial biopsy and suturing simulator will be made of silicone and will not include color information, colors can be omitted during this stage of the process (Fig. 3a). These modeling and sculpting steps are hereafter referred to as processing steps. The design decisions for simulating individual pathological structures and wound configurations were guided by typical morphology encountered in basal cell carcinomas and post-excisional wound shapes. These reference patterns were derived from clinical atlases and consensus teaching materials regularly used in undergraduate dermatology education.

At the end of these processing steps, a colorless model is created featuring two tumor-like lesions intended to represent basal cell carcinomas (the most common form of nonmelanoma skin cancer) and a gaping wound above the upper lip (Fig. 3a). Since this model will be reproduced in silicone, given that equivalent 3D printing materials are both costly and inferior to silicone in terms of tactile properties, a negative mold of the model is created. To achieve this, the positive 3D model was digitally modified in Blender by adding an outer shell of 1 mm thickness via the “solidify modifier”, which generates an additional surface offset at a defined distance from the original model, thereby creating a hollow volume suitable for printing as a mold. Alternatively, the model can also be subtracted from a larger cube to obtain the negative mold. This is done virtually by placing the 3D model inside a cube and applying a Boolean “difference” operation, which removes the model’s volume from the cube and leaves behind a cavity in the shape of the original object. This mold is then used to cast the structure in silicone.

### Printing the 3D model

The completed mold is exported as a stereolithography (.stl) file, the most common file format used for creating 3D models. The .stl file is then opened in a slicer program (Fig. 3b). A slicer is a software tool that makes 3D models executable for 3D printers. A popular choice for this purpose is the free software Cura (Version 5.7.1, Ultimaker Cura, Lesser General Public License (LGPL)v3). The support structures were enabled, and the molds were printed in a horizontal orientation to optimize the surface quality and minimize deformation.

In this instance, printing the model took 36 min and consumed 9 g of material, which, at a material cost of approximately €20 per kilogram, amounts to approximately €0.18. The volume of the mold is approximately 10.47 cm³.

At this stage, we used the FLSun Super Racer [32], a 3D printer employing the Fused Deposition Modeling (FDM) method, where a polylactic acid (PLA) thermoplastic filament is melted at the desired positions with the help of a heated nozzle. The molds were printed via a 0.4 mm nozzle, a layer height of 0.2 mm, and a print speed of 100 m/s. The PLA filament was extruded at 200 °C with a heated bed at 60 °C. The 3D printer features a print head mounted on three movable jointed arms, which move the print head in various directions to change its position. The printer’s arms are driven by a 48-mm stepper motor, allowing the lightweight print head to move quickly, enabling printing speeds of up to 200 mm/s. Using this process, the final mold is produced (Fig. 3c).

### Creating the cast object

**Fig. 4:**
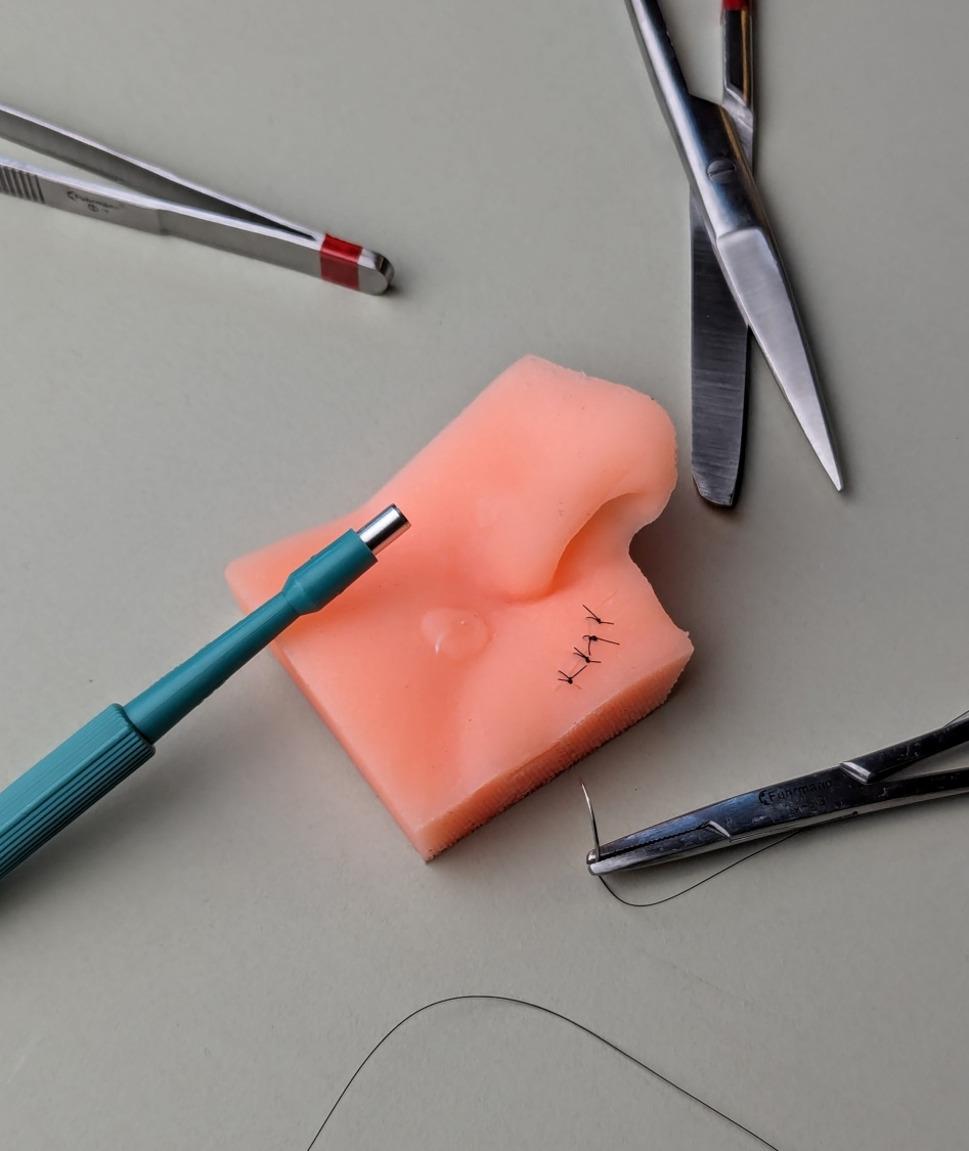
Results of the silicone casting process. 3D skin model molded from silicone for use in the course

The printed mold (Fig. 3c) was filled with two-component room-temperature curing silicone (R PRO 10 molding silicone soft 1:1, Reschimica srl, Florence, Italy) (Fig. 4). The silicone components were mixed in equal parts according to the manufacturer’s instructions, degassed to minimize air bubbles, and carefully poured into the assembled mold until the cavity was completely filled. After being poured, the mold was left to cure at room temperature for several hours before the silicone mold was removed and underwent minor postprocessing, such as trimming excess material with a scalpel and removing filament threads with tweezers. The final models exhibited a homogenous surface without visible layer lines or air inclusions.

Given that the elasticity of human skin is approximately 21 on the Shore-A hardness scale [33], we selected softer silicone with a hardness of 10 A. This lower Shore-A value was chosen because the model was cast as a solid block rather than as a thin skin layer. Using a slightly softer material compensated for this difference and resulted in a more realistic tactile impression during biopsy and suturing practice. Since the skin model is cast as a solid block, the bulk material provides less flexibility and deformation under pressure than a thin silicon sheet does. In other words, even when the same silicone is used, a thick block feels firmer than a thin membrane that mimics only the skin’s surface. This effect was considered when a softer silicone compound was selected to achieve a realistic tactile sensation during training. Each silicone block could be used for approximately 10–15 biopsy and suturing procedures before structural damage (e.g., tearing at incision sites) can occur, making further use impractical. The choice of silicone type and hardness was based on previously published biomechanical studies of human skin elasticity as well as internal pilot testing to determine which material properties best supported the procedural steps defined above. Our selection aimed to balance anatomical realism with durability and reproducibility in repeated teaching scenarios.

The cost of silicone per model is approximately €0.44, which is calculated on the basis of a silicon price of €35/kg, a density of 1.2 g/cm³, and a volume of approximately 10.5 cm³. Printing required approximately 36 min per model, molding for approximately 2 h, and postprocessing for 4 h for all the models. The one-time modeling phase, including the preceding design steps and all iterations, took approximately 30 h for an with 3D technologies experienced user. The material cost including the 3D printed mold was €0.62 per model. This does not reflect the actual production cost, as the time required for 3D design, including iterative development, print file preparation, printer handling, as well as molding and demolding processes, was not included.

### Statistics

Data collection and preparation of the figures were performed via Microsoft^®^ Excel^®^ and Microsoft^®^ PowerPoint^®^ for Mac^®^ 2022 (Microsoft Corporation, Redmond, WA, USA). The students’ feedback on the skin model was gathered through an anonymous online questionnaire, which included items on perceived realism, suitability for practicing punch biopsies and suturing, and the effect on their confidence in performing these procedures on patients. The questionnaire was created via SosciSurvey and made available for evaluation via a quick response (QR) code. The null hypothesis of the study was as follows: Concerning the question “The 3D skin model offers me a realistic opportunity to practice skin biopsies”, there is no difference between the three models. Descriptive statistics and figures were created via R^®^ version 4.3.1 (R Core Team 2022, R Foundation for Statistical Computing, Vienna, Austria) for Mac^®^ [34]. A step-by-step production protocol is available from the corresponding author upon reasonable request, enabling reproducibility at other institutions. No a priori power calculation was performed, as the study was exploratory in nature. Because this pilot study was embedded in a fixed curricular cohort, the sample size was determined by the number of students enrolled in the course and could not be adjusted for statistical power considerations. As a result, the findings should be interpreted as preliminary and hypothesis-generating rather than confirmatory, and the limited sample size may reduce the generalizability and statistical robustness of the results.

## Results

**Fig. 5:**
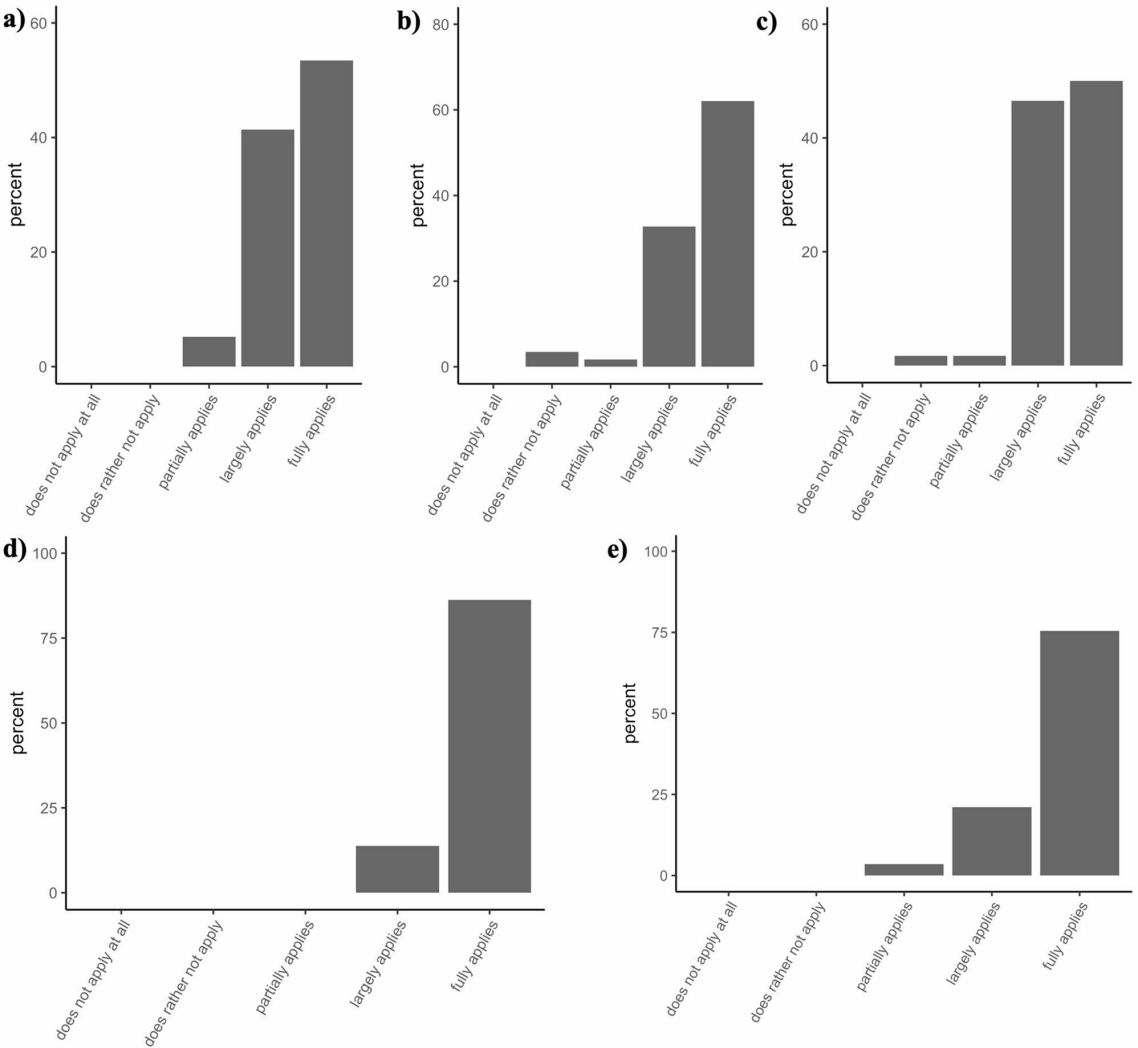
Student evaluation of the 3D skin model. (**a**) The 3D skin model is realistically modeled on real anatomy. (**b**) The 3D skin model offers me a realistic opportunity to practice skin biopsies. (**c**) After practicing the procedure on the 3D model, I feel more confident in my ability to perform this procedure on a patient for the first time. (**d**) I would like to see surgical exercises on 3D skin models become an integral part of dermatological/surgical training in medical studies. (**e**) The 3D skin model helped me practice a trial biopsy with single-button sutures in a safe environment and to prepare for my first procedure on a patient

The project achieved two important outcomes: the successful development of an anatomically realistic 3D training model (Fig. 4) and its implementation in dermatology education, accompanied by student feedback (Fig. 5). To gather feedback, an evaluation questionnaire was developed by the study team and subsequently reviewed and approved by the Ethics Committee of Ludwig Maximilian University of Munich (project number 23–0884). At the end of the course, the students received a QR code linked to an anonymous and voluntary survey. Out of the 82 participants from the winter semester of the year 2023–2024, 58 completed the questionnaire in full, whereas one submitted a partial response.

Key demographic characteristics of the participants are summarized in Table 1. The students expressed great enthusiasm for the silicon-based skin simulator, noting that after repeated practice, they reported greater confidence in performing a skin biopsy on a real patient for the first time. In addition to these positive perceptions, the students emphasized that working with the model within the structured teaching session helped them understand each procedural step more clearly and link the practical exercise to the theoretical content covered beforehand. This alignment with the course’s learning objectives contributed to a stronger understanding of the steps involved in planning, performing, and assessing a punch biopsy. As no baseline measurement of confidence was obtained, this represents a subjective self-assessment rather than an objective comparison. Students were additionally provided with individual suture kits after the course containing all necessary materials for practice, which they could also use outside the course sessions. However, the evaluation questionnaire was completed directly at the end of the course, before extended use of the suture kits at home could have influenced the responses. The students reported that having personal suture kits and the opportunity to practice with the 3D-printed skin model helped them feel better prepared for the objective structured clinical examination (OSCE) at the end of the semester. This perception is consistent with previous reports that structured skills training and simulation-based practice can enhance student readiness for OSCEs [35, 36]. Furthermore, the students highlighted that having personal suture kits allowed them to continue practicing for future surgical procedures.

**Table 1:** Demographic characteristics of the participants

Number of semesters I have fully completed, *n* (%)	
2nd Semester	4 (7%)
3rd Semester	55 (93%)
**I already have previous experience in performing minor surgical procedures on patients independently**, n (%)*	
None	48 (82.8%)
Rather no	2 (3.4%)
Neither Rather yes Absolutely yes	3 (5.2%) 4 (6.9%) 1 (1.7%)

*Only completely assessed datasets were evaluated, *n* = 58. The data are presented as the number of students (n) and percentages (%)

The survey results revealed overwhelmingly positive feedback. A tabular summary of the main findings can be found in Table 2. The students found the 3D skin model to be highly realistic, with 94.8% agreeing in the questionnaire that it closely resembled real anatomy (Fig. 5a). The same percentage regarded it as an effective way to perform skin biopsies (Fig. 5b). Additionally, 96.6% of the respondents reported an increase in their confidence in performing such procedures on real patients after practising on the model (Fig. 5c). As no baseline or comparator group was included, these data represent subjective self-assessments rather than objective measures of competence. They emphasized that the opportunity to practice in a controlled, safe environment helped them prepare for their first patient encounters (Fig. 5e). All the students supported the idea of incorporating 3D biopsy and suturing simulators into standard dermatology and surgical education within medical training programs (Fig. 5d).

**Table 2:** Key evaluation results of the 3D skin model

Evaluation Item	Result	Percentage
Model resembles real anatomy	Agree / Strongly agree	**94.8%**
Provides realistic opportunity to practice biopsies	Agree / Strongly agree	**94.8%**
Increased confidence for first patient procedure	Agree / Strongly agree	**96.6%**
Support integration of 3D models into training	Agree / Strongly agree	**100%**
Model helped prepare safely for first biopsy	Agree / Strongly agree	**96.5%**

## Discussion

The focus of this study was the development and implementation of a cost-effective, anatomically accurate 3D-printed skin simulator to enhance practical medical training, particularly in performing punch biopsies and suturing techniques. Our findings demonstrate that the model enabled realistic procedural practice and was well received by students, supporting its value as an instructional tool. The process of creating a model that meets the requirements for punch biopsy and suturing was iterative and included numerous challenges, such as air bubble formation and difficulties in achieving sufficient mobility of the simulated skin layers. Each of these challenges required innovative solutions, such as modifying the mold design to include a material bridge that allows realistic skin mobility.

The educational relevance of the simulator extends beyond its technical realism. Its integration into a structured skills curriculum enables students to acquire biopsy-related competencies in a safe, scaffolded learning environment. As such, the model supports early clinical preparedness and aligns well with principles of competency-based medical education.

The 3D facial skin simulator’s low material cost (of €0.62 per unit) and its ability to mimic real human skin make it a cost-effective and accessible training tool for large teaching cohorts. These results align with the broader evidence that effective skills acquisition depends less on technological sophistication and more on task-specific realism, functional anatomy, and opportunities for repeated practice [37]. In addition, not all 3D models can be produced cost- and time-effectively, as this depends on whether multiple materials and various techniques are used [8]. However, its reliance on silicone—a material that is not easily recyclable—raises questions about its environmental sustainability, which are admittedly also important when using foam materials or food. Future iterations of the model may benefit from exploring alternative, more sustainable materials while maintaining haptic and visual realism. While materials such as polylactic acid (PLA), which is biodegradable under industrial composting conditions, has limitations when it comes to simulating soft tissues characteristics due to its stiffness, emerging biopolymer composites (e.g., gelatin-, alginate-, or chitosan-based biomaterials) represent promising options [38–40]. These materials may offer improved environmental profiles and, depending on their formulation, could be engineered to mimic the elasticity and multilayer structure of human skin more closely. Their feasibility for producing anatomically accurate, reusable skin models warrants further investigation.

Comparable validation efforts for dermatologic 3D models have been reported in recent literature, including Hammoud et al., who demonstrated the feasibility and educational value of validated 3D-printed skin simulators for procedural training in dermatology [41]. Furthermore, Garg et al. and Dunnick et al. demonstrated that dermatologic 3D simulation models can effectively enhance learners’ diagnostic and procedural competencies, underscoring the broader value of validated simulators in dermatology education [42, 43]. Together, these findings highlight the need for systematic validation of our model to ensure reproducibility, instructional relevance, and long-term integration into dermatologic training programs.

One of the most critical aspects of the model’s success is its adaptability. Importantly, the digital workflow allows adaptation of the model for alternative anatomical regions or advanced procedures without substantial redesign. This adaptability enables broader applications in other medical disciplines, such as general surgery or plastic surgery, where realistic training is equally important. Furthermore, integrating additional features, such as a simulated blood source, could enhance the model’s fidelity and further improve its utility in advanced surgical training.

Despite its potential, the model’s widespread adoption faces logistical and institutional challenges. These include the need for personnel with technical expertise in 3D modeling, printing, and mold preparation, as well as access to suitable printing hardware and materials. In some institutions, initial financial investment, limited staffing resources, or lack of dedicated fabrication facilities may further hinder the scalable implementation of such training models. However, open-source sharing of 3D data and instructions could enable wider accessibility and encourage collaboration among medical schools.

The model’s introduction has already demonstrated a positive impact on medical training, particularly in enhancing student confidence and preparedness for real-life procedures and therefore ensuring patient safety. Nevertheless, future studies comparing fruit and foam models with the newly printed silicone-based skin simulator are essential to enable direct randomized comparisons with established training tools and evaluate teaching outcomes, a process currently underway in our institution. Direct head-to-head comparisons of the 3D biopsy and suturing simulator with existing training materials (such as fruit, foam, animal tissue, or commercial silicone pads) were not within the scope of this study. These analyses are part of a separate investigation currently underway and will be reported elsewhere. This exploratory, single-center pilot study lacks formal validation and comparative controls, which limits generalizability and statistical robustness. Manning et al. also reported that early and prolonged opportunities to practice suturing, such as during cadaver dissection, increased the students’ comfort with suturing during clerkship [44]. However, its focus on a specific task - punch biopsy - raises questions about its sufficiency as a standalone training tool. Expanding its applications to include a wider range of procedures, e.g., complex flap reconstructions, will likely be necessary to maximize its value in medical education. Furthermore, working with and gaining experience using 3D printed models increasingly equips students for real-world clinical practice, where patient-specific 3D models, such as those of organs, are already being utilized for surgical operation planning [45].

While this study highlights the potential of the 3D-printed skin simulator as a cost-effective and anatomically accurate tool for medical training, it is important to acknowledge several limitations. However, as this was an exploratory pilot study, the model has not yet undergone formal validation, and the results should therefore be interpreted with caution. First, the study focused exclusively on training for punch biopsies and simple interrupted sutures, with no head-to-head comparisons conducted for other surgical procedures, such as spindle excisions or transposition flap reconstructions. This limits the generalizability of the model’s utility across a broader range of dermatological and surgical techniques. Second, no baseline measurement or comparator group were included, meaning that all outcomes reflect subjective student perceptions rather than objective performance improvements. In particular, the study did not incorporate a direct comparison with other high- or low-fidelity training models, such as animal tissues or commercially available synthetic materials, making it difficult to definitively evaluate the 3D model’s relative effectiveness. The lack of randomized controlled trials to assess teaching outcomes further reduces the ability to draw robust conclusions about its superiority. This was primarily due to the fact that the sample size was fixed by the curricular cohort and therefore not powered for statistical inference. Another limitation is the absence of features such as simulated bleeding or varied tissue textures, which could enhance the model’s realism and applicability for more complex procedures. Furthermore, while the silicone material used is cost-effective and realistic, it may not be sufficiently environmentally sustainable, raising concerns about the long-term ecological impact of widespread implementation. In this study, students were also provided with individual suture kits for additional practice at home. While this measure was didactically valuable and may have supported further skills training, it also represents a potential source of bias, since improvements in confidence and skills cannot be attributed solely to the use of the artificial training model. Nevertheless, the evaluation was conducted immediately at the end of the course; therefore, the survey results primarily reflect the direct training effect with the 3D model rather than prolonged unsupervised practice, which may limit insights into long-term skill retention. Finally, the study’s reliance on advanced 3D printing technology and technical expertise may pose barriers to its implementation in resource-limited institutions. However, if the model design and process are openly available, the practical challenges of replicating the production process could hinder broader dissemination.

Addressing these limitations in future iterations and studies will be essential to validate the 3D skin model’s effectiveness, expand its range of applications, and promote its widespread use in medical education.

Finally, the project’s success underscores the importance of iterative development, interdisciplinary collaboration, and alignment with curricular learning objectives. As medical education continues to evolve, innovations such as the silicone-based skin simulator highlight the potential of technology to enhance hands-on learning experiences, improve patient safety, and prepare future healthcare professionals for clinical practice.

## Conclusions

In conclusion, the development of the 3D skin model represents a significant improvement in medical education, particularly in teaching punch biopsies and suturing. This realistic, cost-effective, and reusable model addresses the shortcomings of traditional training materials, such as foam and fruit, which lack the necessary haptic realism and anatomical accuracy.

Student feedback has been overwhelmingly positive, with participants reporting increased confidence and preparedness for performing real clinical procedures. The model’s low production cost and high usability make it a scalable solution for medical training.

The success of this project highlights the potential of innovative tools such as the 3D-printed skin simulator to enhance hands-on learning and improve clinical competency, setting a new standard for practical training in medical education. Future studies are crucial to compare conventional models with the newly printed 3D biopsy and suturing simulator, facilitating direct comparisons and further assessment of teaching outcomes, a process already in progress.

## Data Availability

The data are available from the corresponding author upon reasonable request.

## Abbreviations

3D: Three-Dimensional
FDM: Fused Deposition Modeling
NKLM: National Competency-Based Learning Objective Catalog
OSCE: Objective Structured Clinical Examination
PLA: Polylactic acid
QR: quick response
GPL: General Public License
LGPL: Lesser General Public License
STL: Stereolithography
